# Immune-mediated cytopenias (IMCs) after HSCT for pediatric non-malignant disorders: epidemiology, risk factors, pathogenesis, and treatment

**DOI:** 10.1007/s00431-023-04912-6

**Published:** 2023-03-27

**Authors:** Manuela Spadea, Francesco Saglio, Valeria Ceolin, Marta Barone, Giulia Zucchetti, Paola Quarello, Franca Fagioli

**Affiliations:** 1 Stem Cell Transplantation and Cellular Therapy Department, Pediatric Onco-Hematology, Azienda Ospedaliera-Universitaria Città Della Salute E Della Scienza, Regina Margherita Children’s Hospital, Turin, Italy; 2 University of Torino, Turin, Italy; 3 Erasmus University MC-Sophia Childrens Hospital, Rotterdam, Netherlands

**Keywords:** Pediatric inherited disorders, Pediatric stem cell transplantation, Immune-mediated cytopenias, Children

## Abstract

Hematopoietic stem cell transplantation (HSCT) represents a curative option for pediatric patients affected by malignant and non-malignant disorders. Several complications may arise during the post-transplantation period, including immune-mediated disorders. Immune-mediated cytopenias (IMCs) account for up to 22% of pediatric HSCT complications, representing an important cause of morbidity and mortality post-HSCT. So far, their pathogenesis is not well-understood, and their management may be very challenging. Further, most patients are refractory to first-line treatment which is based on high-dose intravenous steroids, immunoglobulin, and the monoclonal anti-CD20 antibody — rituximab. No clear consensus has been reached for second- and third-line therapeutic options.

*Conclusion*: We reviewed the epidemiology, risk factors, pathogenesis, and treatment of IMCs, aiming to offer a deeper understanding of these complications as a guide to improving the management of these fragile patients and a cue for the design of tailored clinical trials.

**Table:** 

**What is Known:** *• IMCs arising in the post-HSCT setting represent a rare but potentially life-threatening complication. Younger patients affected by non-malignant disorders are at the greatest risk of IMCs arising after HSCT. Corticosteroids, intravenous immunoglobulin, and rituximab represent the undiscussed first-line therapeutic approach.*	
**What is New:** *• This review highlitghts how children present unique risk factors for post HSCT IMCs, which are the result of the complex relationship between the immaturity of their infantile immune system and all the perturbing agents and factors which characterize the post-HSCT setting. Future efforts are warranted to establish the best option for refractory patients, for whom a standard and validated approach is not currently available. Among new agents, ibrutinib or bortezomib and fostamatinib or low-dose IL-2 could represent a good therapeutic option for patients with graft-versus-host disease and hemolytic anemia or graft-versus-host disease and thrombocytopenia, respectively.*

**What is Known:**

*• IMCs arising in the post-HSCT setting represent a rare but potentially life-threatening complication. Younger patients affected by non-malignant disorders are at the greatest risk of IMCs arising after HSCT. Corticosteroids, intravenous immunoglobulin, and rituximab represent the undiscussed first-line therapeutic approach.*

**What is New:**

*• This review highlitghts how children present unique risk factors for post HSCT IMCs, which are the result of the complex relationship between the immaturity of their infantile immune system and all the perturbing agents and factors which characterize the post-HSCT setting. Future efforts are warranted to establish the best option for refractory patients, for whom a standard and validated approach is not currently available. Among new agents, ibrutinib or bortezomib and fostamatinib or low-dose IL-2 could represent a good therapeutic option for patients with graft-versus-host disease and hemolytic anemia or graft-versus-host disease and thrombocytopenia, respectively.*

## Introduction

Hematopoietic stem cell transplantation (HSCT) represents the main therapeutic option for several non-malignant disorders, including bone marrow failure syndromes, primary immunodeficiencies, and inborn errors of metabolism. The delicate immune balance characterizing immune reconstitution post-HSCT [1] could be disrupted by the development of immune-mediated cytopenias (IMCs) [2]. This is particularly evident in pediatric patients transplanted for non-malignant diseases, in whom IMCs represent an important cause of morbidity and mortality post-HSCT [3]. However, the increasing awareness, prompt recognition, and a broader range of therapeutic approaches have led to improving survival in this cohort of patients [3]. We reviewed current evidence in epidemiology, risk factors, pathogenesis, and available treatment options for these complications with the aim to exploit a better understanding of these potentially life-threatening complications for a more accurate clinical management and better outcomes.

## Epidemiology and risk factors

IMCs occurring post-HSCT in patients affected by non-malignant diseases have a reported incidence that varies widely through literature: from a 1.5 to 22% of pediatric reports [4–17]. IMCs may manifest as hemolytic anemia, thrombocytopenia, and neutropenia, alone or in combination (e.g., Evans syndrome, which results from the association of immune hemolytic anemia and thrombocytopenia or neutropenia). The median reported time to develop IMCs is approximately 2 to 40 months post-HSCT, regardless of the ongoing immunosuppression for prophylaxis or treatment of graft-versus-host disease (GvHD) [4–10, 18, 19]. Hemolytic anemia remains the most frequent form of IMC [2, 6, 8–10, 17, 18, 20, 21]. Immune thrombocytopenia has been reported in 0.5–2% of pediatric HSCT patients [3, 6, 14]. Immune neutropenia is rare, and no data about its incidence is available to date [22]. Risk factors related to IMCs post-HSCT might be classified in patient-specific, donor–recipient HLA matching-related, and transplant-associated [3] (see Table 1). For the first group, patient age and the underlying disease appeared to have the utmost influence on the development of IMCs [3]. Particularly, a greater incidence of IMCs has been highlighted in several reports among younger patients [2, 5–10, 17–19, 23] and in patients transplanted for non-malignant diseases (especially primary immunodeficiencies and inborn errors of metabolism) [4, 6, 9, 10, 20, 23, 24]. It could be stated that being transplanted for inherited disorders represents per se a risk factor for developing IMCs post-HSCT [23]. Interestingly, a recently published retrospective single-center study found pediatric patients transplanted for hemoglobinopathies as having an increased 1-year cumulative incidence of IMCs [25]. This suggests that previous alloimmunization consequent to multiple transfusions might somehow contribute to the development of these complications [26], even if it is difficult to demonstrate its pathogenicity, because of the multiple concurrent factors increasing the risk in this cohort of patients. Moreover, Szanto et al. demonstrated that chemo-naivety presented a stronger relationship (*P* = 0.004) than “non-malignant indication” (*P* = 0.02), highlighting that the chemo itself prevents the development of IMCs, perhaps because chemotherapy removes the immune cells involved in IMCs development, prior to HSCT [15]. From a donor–recipient compatibility standpoint, the choice of unrelated donors, particularly if mismatched, and haploidentical ones have been related to the development of these complications [4, 6, 13, 27]. Finally, the HSCT procedure itself and its following complications might increase the risk of immune cytopenias [2]. Indeed, the use of lymphodepleting agents to prevent GvHD during the conditioning regimen has been reported in several pediatric reports as a crucial risk factor (alemtuzumab > ATG) [2, 9, 13, 15, 26]. Besides, the conditioning choice may increase the risk for IMCs: this has been demonstrated in patients affected by severe aplastic anemia, in whom the use of reduced intensity conditioning was associated with the development of post-HSCT immune complications [14, 26]. Indeed, it has been postulated that the development of mixed chimerism (that often follows a reducedintensity conditioning regimen and is more tolerated, as well as found, after HSCT for a non-malignant indication) could further increase the risk of IMCs after HSCT, even if this point is still a matter of great debate (9,12,15). Some pediatric reports are identified as a risk factor even the source of stem cells (UCB > PBSC > BM), but multivariate analysis did not confirm the findings [6, 14, 15, 17–19, 26, 28]. Likewise, the development of GvHD seemed to increase the risk of immune complications, but only a single-center retrospective study was able to classify aGvHD grades II–IV as an independent risk factor for IMCs in the pediatric population [15]. Interestingly, this study was also able to underline that pediatric patients treated with serotherapy, chemo-naïve prior to HSCT, and who developed aGVHD grades II–IV before IMC development had a 22% chance to develop immune cytopenias post-HSCT [15]. Furthermore, chronic GvHD has been reported as one of the key risk factors for developing IMCs, even for pediatric patients [2]. Finally, infectious complications, particularly viral reactivations, could act as a trigger for the development of IMC post-HSCT during pediatric age [2, 26]. This has been highlighted especially for CMV [9], which has been recognized as one of the primary risk factors.

**Table 1:** Risk factors associated to post-HSCT IMC development in pediatric patients affected by non-malignant disorders

Patient-specific	Donor–recipient HLA matching-related	Transplant-associated
Younger age at HSCT Non-malignant disease (especially primary immunodeficiencies and inborn errors of metabolism) Chemo-naivety prior to HSCT	Matched and mismatched unrelated donors Haploidentical donors	Use of lymphodepleting agents to prevent GvHD (alemtuzumab > ATG) Reduced intensity conditioning Source of stem cells (UCB > PBSC > BM) aGvHD grades II–IV cGvHD Viral infections (particularly CMV) Mixed chimerism

## Pathogenesis

**Fig. 1:**
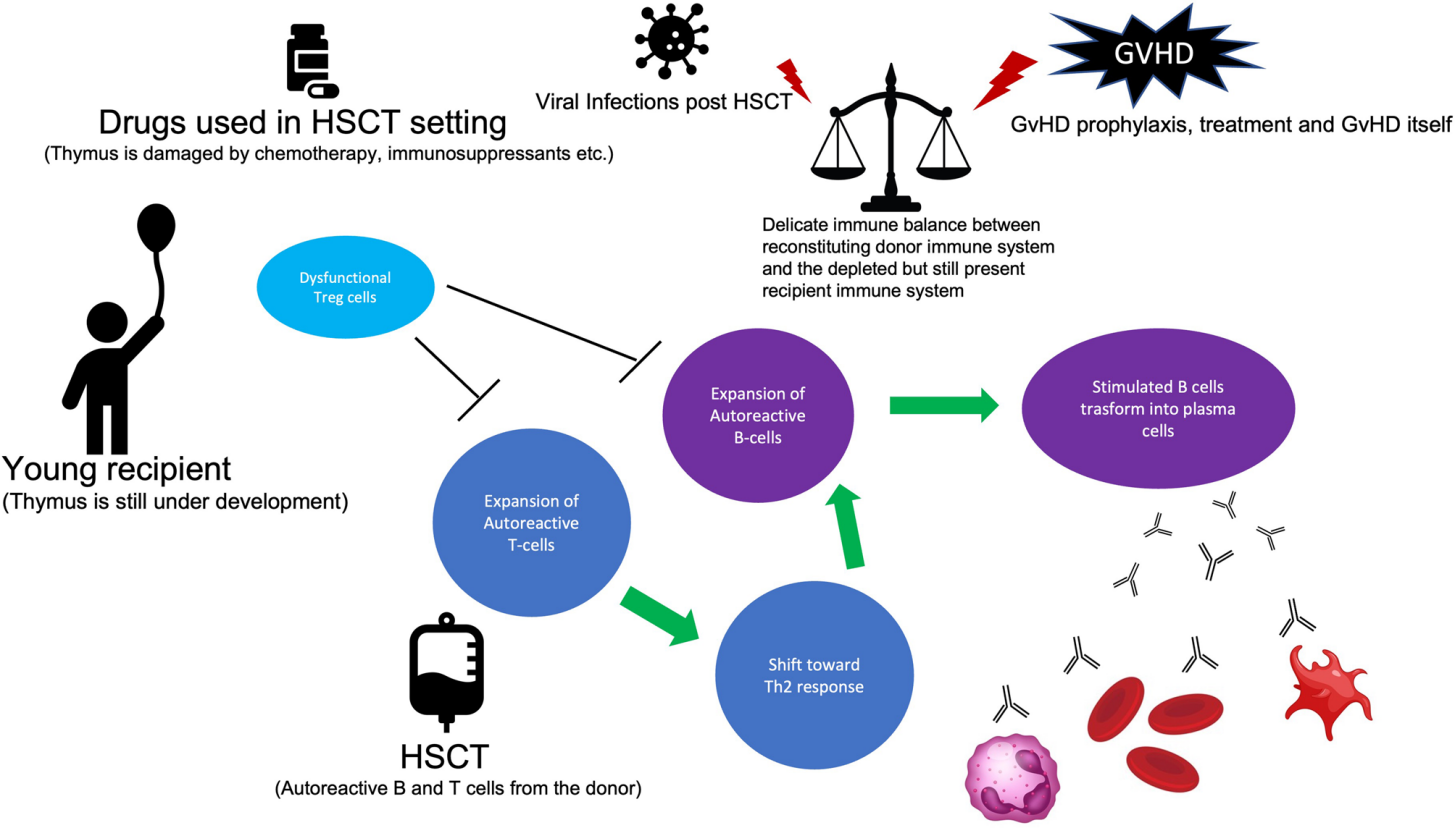
Pathogenesis of IMCs post-HSCT. Different pathomechanisms have been proposed: an impaired central tolerance due to thymic damage arising from chemotherapy or immunosuppressant drugs employed in HSCT setting or related to the immaturity of the thymus in young children; the donor could transfer to the recipient autoreactive B and T cells; and the delicate immune balance between the reconstituting donor immune system and the depleted but still present recipient immune system could be shifted toward aberrant immune responses — by graft-versus-host disease and its related therapies or prophylaxis or by viral reactivations. All these factors could lead toward the expansion of autoreactive T and B cells and toward the shift to a T helper 2 response which could amplify itself the expansion of autoreactive B cell and their transformation into plasma cells producing antibodies that definitely recognize antigens present on red blood cells, platelets, or neutrophils

The mechanisms behind the development of IMCs post-HSCT are not completely understood (see Fig. 1). This is because of the unique picture characterizing the post-transplantation period, in which a delicate balance between the reconstituting donor immune system and the depleted but still present recipient immune system is crucial for avoiding immune complications [1]. In this scenario, it is of paramount importance a prompt reconstitution and appropriate functioning of T regulatory cells (Tregs) which suppress autoreactive T and B cells, ultimately preventing their expansion which leads to consequent aberrant immune responses [2, 3]. Indeed, a paucity of Tregs is characteristic of younger patients in whom the thymus is still under development [2, 3, 7, 17] and, broadly, of the early post-HSCT setting (because the thymus is damaged by the conditioning regimen) or late post-HSCT setting (in which additional complications, such as GvHD and its related treatments, could further damage the thymus) or, finally, of the T-deplete haploidentical setting [29]. Similarly, patients with IMCs showed inadequate Tregs’ capabilities in controlling T helper cell type 2 (Th2) activation, which ultimately resulted in Th2-driven autoimmunity [9]. Besides Tregs/Th2 imbalance, pediatric patients presenting IMCs showed also aberrant immune reconstitution profiles, with a trend toward lower NK, CD3 + CD8 + cells, and higher values of IgM at the time of IMC onset [15]. Taken all together, these data confirm that both T cell and B cell dysfunction lay the foundation for the development of immune cytopenias, and it is quite challenging to distinguish auto- from allo-immunity in this context. Thus, the definition of “immune cytopenias” better fits these complications. Besides the proposed mechanisms, the donor could cause the transfer of autoantibodies and autoreactive B and T cells, or both, into the recipient, triggering the development of IMCs [22]. Furthermore, taking advantage of lessons learned from patients affected by inborn errors of immunity, in whom several immune mechanisms breaking immune tolerance have been proposed [30], we could also speculate that other pathomechanisms could be implied in autoreactive B and T cells expansion in patients with IMCs post-HSCT. Indeed, from a T cell standpoint, aberrant T cell receptor signaling could compromise central tolerance by affecting negative selection of autoreactive T cells, while defective lymphocyte apoptosis could lead to a failure in controlling lymphocyte expansion in the context of an adaptive immune response [30]. On the other hand, the central B cell tolerance, which is deployed within the bone marrow and is achieved through several mechanisms (e.g., anergy, clonal ignorance, clonal deletion, and receptor revision), could be incomplete leading to a considerable proportion of autoreactive B cells escaping the bone marrow [30]. Considering the limited information on these variables, further studies are needed for a thorough exploration of the interconnection of T cell- and B cell-mediated pathomechanisms.

In this context, it has to be considered that all the factors which influence the speed and quality of donor immune reconstitution may lead toward IMCs’ development: the underlying disease, the use of lymphodepleting agents (ATG or alemtuzumab), the source of the stem cells, the choice of a mismatched donor, the development of GvHD, and the administration of calcineurin inhibitors to prevent GvHD or infectious complications [1, 31]. This reflects the known reported data about the relationship between the cited variables and IMCs’ development.

Finally, we could speculate that all the cited factors are interrelated in children affected by non-malignant disorders as compared to those with hematological malignancies who undergo HSCT. As explained above, these patients are relatively younger (with a still immature and under development thymus), and they also have an underlying immune system that is presumably intact because of no prior exposure to chemotherapy. In some of them presenting with metabolic diseases, even lysosomal glycosaminoglycan accumulation could be implicated in innate and adaptive immune system dysfunction, while others with hemoglobinopathies (especially those affected by thalassemia) could present an alloimmunization consequent to multiple transfusions which could eventually hamper post-HSCT immune dysregulation.

## Diagnosis

IMCs could be related to several causes when they occur in the post-HSCT setting. Hence, important SCT-related complications which usually present with overlapping features should be ruled out before considering IMCs: graft-versus-host disease, hemolytic anemia owing to ABO blood group incompatibility, transplantation-associated thrombotic microangiopathy, graft failure, disease relapse, active infections, side effects of drugs, and disseminated intravascular coagulation.

A stepwise diagnostic approach is recommended [22], starting from a proper review of the transplantation procedure itself (evaluating patient-specific, donor–recipient HLA matching-related and transplant-associated risk factors).

First-line diagnostic tests include differential blood count with reticulocytes and a blood smear, chemistry including creatinine and hemolytic parameters (i.e., direct and indirect bilirubin, lactate dehydrogenase, and haptoglobin), and urinalysis. It is worth remarking that very young infants could falsely show decreased haptoglobin because they often have poor synthesis capabilities for this protein [3].

In case of hemolytic anemia, a direct antiglobulin test (DAT) is mandatory, and it is often positive in patients with post-HSCT autoimmune hemolytic anemia, even if a negative DAT does not rule out the condition [32]. DAT usually reveals complement (C3) and/or immunoglobulin (IgM or IgG) antibodies on the surface of red blood cells. Indirect Coombs testing may also yield the presence of serum antibodies.

In case of immune thrombocytopenia and neutropenia, anti-human platelet antigen or anti-human neutrophil antigen antibodies may be present on testing, but their presence does not exclude other causes of these cytopenias, and their absence does not exclude a diagnosis of an immune-mediated destructive process [15]. Indeed, the relatively low sensitivity of anti-platelet and anti-neutrophil antibody testing compared to that for immune hemolytic anemia adds another hurdle to diagnose multiple lineage cytopenias or isolated immune thrombocytopenia and neutropenia. For both platelet and neutrophil antibody testing, the earlier techniques, ELISA-based, have low specificity and sensitivity (60–70%) [23]. The more recent assays, often flow cytometric immunobead assays, are only marginally better, reaching a sensitivity and specificity of about 80% [33, 34]. For these reasons, the diagnostic criteria for immune thrombocytopenia and neutropenia could not be routinely and exclusively based on the presence of antibodies alone. Further, in case of thrombocytopenia, a pattern of transient response or refractoriness to platelet transfusions could help corroborate diagnosis, while a trial with granulocyte colony-stimulating factor (G-CSF) administration could help confirm immune neutropenia (poor or no response is expected) [22]. A bone marrow aspirate and biopsy should be considered to evaluate chimerism and confirm normal megakaryopoiesis in case of immune thrombocytopenia, while islands of neutrophil precursors and occasionally clear evidence of myeloid maturation arrest are seen in patients presenting immune neutropenia.

## Treatment

There are no guidelines for IMCs’ treatment post-HSCT; therefore, therapy is usually performed following the treatment of immune cytopenias presenting in non-transplanted patients. However, children undergoing HSCT who develop IMCs post-HSCT usually present refractory forms of cytopenias, and second- or third-line therapies are often required.

### First-line treatment

First-line treatment is based on transfusions (even if most patients may be transfusion refractory, because of antibodies in their serum), G-CSF administration in case of neutropenia (isolated or associated with involvement of other cell lines), and hemodynamic supportive therapy. A careful approach must be maintained, such as these complications could present as medical emergencies (especially hemolytic anemia and thrombocytopenia) or could rapidly evolve toward life-threatening situations in such fragile patients. Attention must be paid to transfusion indications: these should be reserved only for patients presenting clinical symptoms of the underlying cytopenia rather than on Hb level or platelet number. This is because autoantibodies could target even the best-matched unit and may contribute to activating further the immune-mediated destructive process. A very few patients — those with the mildest form of isolated neutropenia — might improve solely with this approach, and they would not require any additional agent. However, in most cases, exclusive supportive therapy is not sufficient. In these patients, high-dose corticosteroids are the universal first-line approach (methylprednisolone 2 mg/kg/day until transfusion requirement is < q7 days and then carefully tapering almost over 8–10 weeks) usually associated with intravenous immunoglobulin (0.8–1 g/kg IV daily × 3 days and then consider weekly to maintain goal, tapering based on response), especially in patients affected by immune thrombocytopenia. It is worth remarking that Koo J et al. reported that children with post-HSCT hemolytic anemia had a higher incidence of complications related to steroid treatment (e.g., avascular necrosis and cataracts) as compared to adult patients, and this has to be taken into account when considering the overall duration of steroid treatment [11]. In terms of response to the first-line approach, for patients affected by post-HSCT hemolytic anemia, overall response rates (ORR) vary among reports, from 10 to 90%; however, complete response (CR) with corticosteroids only is generally achieved in 30% or less of cases [26]. Overall, we may state that only one-third of these patients respond to first-line treatment, whereas the remaining two-thirds would require additional agents.

Rituximab, an anti-CD20 chimeric monoclonal antibody that depletes CD20-expressing B cells, has been used both as first-line besides steroid therapy in high-risk patients or as early second-line therapy when a faster steroid tapering is required, following the principle “the sooner the better” [18] (375 mg/m^2^ dose × 4 doses q7 days) and, ultimately, as second-line treatment. The benefit of rituximab addition in pediatric patients was especially highlighted by Faraci et al. who reported an improvement in IMC outcomes with CR of 100% in first-line and 87% in second-line administration [6]. Indeed, rituximab showed encouraging results in literature reports, with an ORR ranging from 66 to 100% and CR ranging from 22 to 100% as well as administered as a first- or second-line agent with a good profile of safety and efficacy. The main complication experienced while using rituximab was hypogammaglobulinemia, with the majority of patients needing immunoglobulin replacement at a median time of 10.5 years after this therapy (range 2.6–15.2 years) [13].

### Treatment beyond first-line therapy

Treatment of refractory patients with second-line agents did not receive full consensus, and clear guidelines for the management of these patients are not always available. So far, several agents have been used. First, various immunosuppressant agents have been added to first-line therapy: sirolimus, mycophenolate mofetil, and azathioprine. It is worth remarking that among patients with hemolytic anemia, sirolimus showed excellent results, with all reported patients developing partial (1 patient) or complete response (10/10 patients from three different case series reported between 2018 and 2020). In patients with persistent severe thrombocytopenia following HSCT, thrombopoietin agonists (e.g., eltrombopag and romiplostim) have also been used and reported achieving a successful response rate, although response takes several weeks [35–38]. Patients presenting immune cytopenias have been treated also by plasmapheresis, but this has been used especially in the acute setting when a cold antibody-mediated hemolysis is suspected, as an additional treatment, while waiting for the response to immunosuppressive therapy [39]. Moreover, cytotoxic agents such as cyclophosphamide could be considered as a salvage therapy if no other options are available [26]. Usually, a regimen based on various combinations of the above-cited agents is preferred. From this standpoint, therapy should be optimized by taking advantage of myeloid growth factors and thrombopoietin and erythropoietin agonist administration, with the aim of reducing to the absolute minimum the transfusion support. For sake of completeness, it should be mentioned that splenectomy is another option; however, it should be considered only in severe forms after the failure of other medical treatments, because of the lower response rate registered in post-HSCT immune cytopenias [26]. From a transplant specialist’s point of view, in patients with mixed chimerism and IMCs, the use of donor lymphocyte infusion or a second HSCT could represent an alternative, but limited data about their effectiveness are available to date [9, 13, 16, 40].

### New agents and ongoing trials for non-responders

Bortezomib is a proteasome inhibitor that has been used to target plasma cells in patients with post-HSCT autoimmunity and confirmed laboratory antibodies and in patients affected by cGvHD [41]. Likewise other pharmacological approaches, few data are available about its beneficial effect in this cohort of patients, but clear conclusions cannot be drawn, since only case reports or small case series have been described with a high risk of reporting bias [42–44]. Two new drugs, daratumumab, an anti-CD38 monoclonal antibody, and abatacept, a fusion protein that modulates the T cell co-stimulatory signal that is mediated through the CD28-CD80/86 pathway, have been used in pediatric patients with refractory autoimmunity with interesting results in some published case series [11, 45–47]. However, no clinical trials are currently ongoing to better clarify their efficacy and safety. For the sake of completeness, alemtuzumab, ofatumumab, and eculizumab have also been used in rare cases, without efficacy [26]. A new option for refractory patients is represented by ibrutinib, a Bruton’s tyrosine kinase (BTK) inhibitor, which has already been used for immune cytopenias presenting in patients affected by chronic lymphocytic leukemia (CLL) [48, 49] and for treating chronic GvHD [50, 51], also in pediatric patients [52], and is nowadays been testing in pediatric patients affected by hemolytic anemia (NCT04398459), even if, so far, not in the post-HSCT setting. Further, a monoclonal antibody, orilanolimab (SYNT001), that blocks the interaction between the neonatal crystallizable fragment receptor (FcRn) and the Fc portion of IgG, ultimately increasing clearance of IgG, has been investigated for its safety and efficacy in adult patients with immune hemolytic anemia (NCT03075878), even if so far there are no available trials for pediatric patients. On the other hand, new promising drugs have been approved in adult patients affected by IMCs post-HSCT, such as fostamatinib — a tyrosine kinase inhibitor — for immune thrombocytopenia, and clinical trials should be designed to extend their administration also to the pediatric population, especially considering that this drug is already been studying in HSCT patients affected by cGvHD (NCT02611063). Finally, another therapeutic option against immune thrombocytopenia could be represented by low-dose IL2, which preferentially induces Treg expansion in vivo and has already been used in some adult patients with thrombocytopenia [53] and for steroid-refractory cGvHD [54]. Furthermore, low-dose IL2 has been used in several pilot studies and clinical trials, even some randomized trials, in patients affected by different autoimmune diseases (including but not limited to systemic lupus erythematosus, type 1 diabetes, rheumatoid arthritis, and different forms of vasculitis) showing a good safety and efficacy profile, independent of the underlying disease [55].

## Unmet needs and future perspectives

**Fig. 2:**
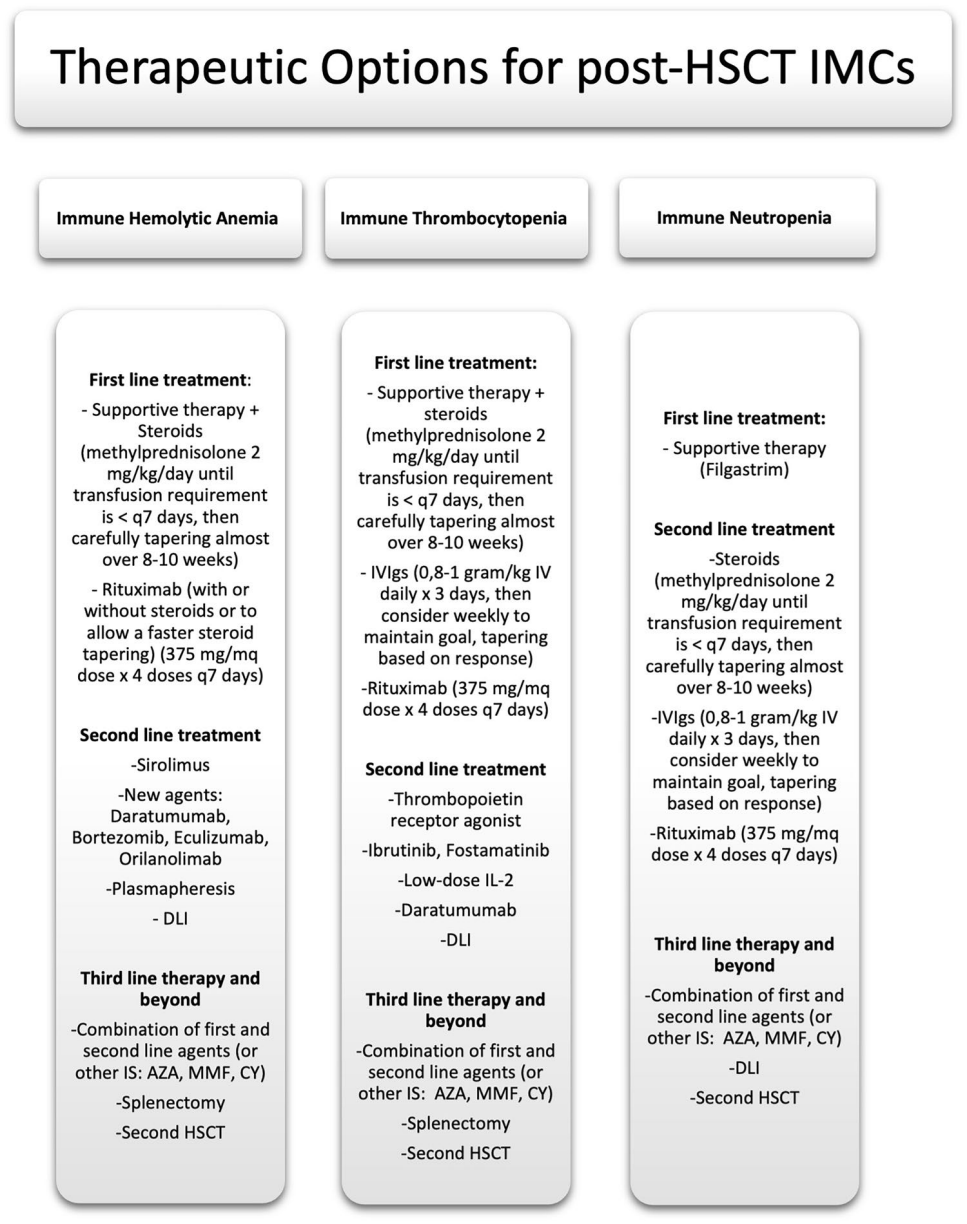
Summary of available therapeutic options for IMCs arising post-HSCT

The greatest challenge is to make conclusions from currently available reports (see Table 2 for a summary of the most relevant cited studies and Fig. 2 for a compendium of therapeutic options). Indeed, most of the available treatments have been reported in single case reports or very small case series, so no evidence supports their administration in treating patients with IMCs post-HSCT. Further studies, possibly perspective or even better randomized controlled trials, are needed to address this question. Moreover, preclinical studies should be devoted to correctly assessing the pathogenesis of IMCs arising post-HSCT, with the aim of developing tailored approaches in this unique cohort of patients. The aim of clinicians should be to enroll refractory patients in designed clinical trials aiming to search for the best therapeutic options in a controlled setting.

**Table 2:** Summary of relevant studies about post-HSCT IMCs in pediatric patients affected by non-malignant disorders

Study	Population	Type of IMCs; incidence	No. of patients with non-malignant disease presenting IMCs	Treatment strategies	Outcome	Comments
O’Brien et al. [4]	439 consecutive pediatric patients undergoing allogeneic HSCT	AHIA; 19/439 patients, cumulative incidence at 1 year 6%	16 patients out of 19 with underlying NMD (11 storage disorders, 5 other non-malignant diseases)	MP (18/19), dex (7/19), IVIg (10/19), CSA withdrawn (8/19), RTX (3/19), PE (2/19), MMF (2/19), EPO (2/19)	10/19 died, 3/10 because of massive hemolysis	Age < 10 years and metabolic disorders confer a higher risk of IMCs post-HSCT Patients developing AIHA were twice as likely to die when compared with patients who did not develop AIHA (RR 2.0, 95% CI 0.9–4.6, *P* = 0.10)
Page et al. [17]	19 consecutive infants (< 3 months of age) undergoing UCB	AIHA, ITP, neutropenia alone or in combination; 10/19 patients, overall cumulative incidence of 44% (95% CI 21–68%) and 56% (95% CI 32–80%) at 1 and 2 years	10 patients affected by NMD (9 early infantile lysosomal storage diseases, 1 beta-thalassemia major)	MP (10/10), RTX (8/10), CSA withdrawn (5/10), IVIg (4/10), azathioprine (7/10), splenectomy (1/10), EPO (1/10), G-CSF (2/10)	1/10 died of multisystem organ failure; OS in the entire cohort of 95% at 1 year	Markedly increased rate of posttransplant autoimmune cytopenias, probably because of immune dysregulation associated with GVHD prophylaxis during the first year of life
Daikeler et al. [5]	726 UCB recipients reported to EUROCORD	AIHA, ITP, neutropenia alone or in combination; 41/52 patients presenting AD post-HSCT had IMCs	26/52 patients affected by non-malignant disorders (6 SAA, 6 SCID, 2 histiocytosis, 12 metabolic disorders)	MP (7/41), RTX (33/41), IVIg (9/41), azathioprine (2/41), CSA added to RTX (2/33), CSA alone (6/41), PE (1/41), no treatment (1/41)	The estimated 5-year OS was 91% ± 9% for patients who developed ITP, 59% ± 11% for those with AIHA, and 67% ± 16% for those with Evans syndrome	All IMCs in this study occurred in cord blood recipients having achieved at least mixed chimerism Non-malignant diseases and interval from diagnosis to CBT < 11.4 months independently associated with the occurrence of ADs after UCB in multivariate analysis
Faraci et al. [6]	1574 pediatric alloHCT	AIHA, ITP, neutropenia alone or in combination; 33/1574 patients presenting IMCS: 15 AHIA (45%), 10 ITP (30%), 5 Evans’ syndrome (15%), 2 pure red cell aplasia (6%), and 1 immune neutropenia (3%). The cumulative incidence of IMCs was 1.53% (95% CI, 1.02 to 2.30) 1 year after HSCT	22/33 patients affected by non-malignant disorders (2 HLH, 1 Langh His, 6 MPS1, 4 SAA, 3FA, 1CA, 2 SCID, 1 WAS, 1 thalassemia major, 1 osteopetrosis)	Corticosteroids (25/33), IVIG (15/33), rituximab (15/33), sirolimus (2/33), EPO (2/33), plasma exchange (1/33)	Four patients (9%) died at a median of 87 days after IMC diagnosis	Use of alternative donor and non-malignant disease statistically associated to IMCs in multivariate analysis RTX represents an efficacious treatment for patients with steroid-refractory disease (87% of CRs in treated patients, 100% in AHIA)
Ahmed et al. [16]	500 pediatric HSCT recipients	AHIA; 12/500 (2.4%) recipients of first HSCT, 7/ 72 (9.7%) recipients of second HSCT	5/12 non-malignant disorders (2 SCID, 1 thalassemia, 1 metabolic disorder, 1 SCAEBV); 4/7 non-malignant disorders (1 thalassemia, 1 SAA, 1 BDS, 1 SCID)	IVIG (5/12 and 4/7), steroid therapy (6/12 and 6/7), Rituximab (5/12 and 1/7), cyclophosphamide(1/12), danazol (2/12); 4 patients received second HSCT to control the AIHA and 3/4 were refractory to the second HSCT	The overall survival did not differ significantly among recipients of single HSCT with and without AIHA	A strong association between the onset of AIHA and HLA-mismatched status was found
Kruizinga et al. [19]	531 pediatric HSCT	AIHA, ITP, neutropenia alone or in combination; 26/531 patients; 3-year cumulative incidence of 5.0% (95% confidence interval, 3.4% to 7.3%)	22/26 non-malignant disorders (9/26 β-thalassemia patients)	Steroids (19/26), IVIG (16/26), rituximab (15/26), wait-and-see approach (6/26), bortezomib (7/26) sirolimus(3/26) splenectomy (1/26) PE (1/26) stem cell boost/second HSCT (4/26)	After a median follow-up of 48 months, there was no significant difference between IMC and non-IMC patients (*P* = .887)	CMV reactivation (hazard ratio, 3.4; *P* = .02), non-malignant diagnosis pre-SCT (hazard ratio, 3.5; *P* = .031), and alemtuzumab use (hazard ratio, 2.5; *P* = .028) were independently associated with the occurrence of AIC Wait-and-see approach was sometimes chosen for non–life-threatening AIC and was efficacious in 83% of these selected patients β-thalassemia patients could be sensitized to the development of AIC because of a history of multiple blood transfusions
Deambrosis et al. [10]	36 UCBT recipients	AIHA, ITP, neutropenia alone or in combination; 8/36 (22%) patients	8/36 affected by Hurler syndrome	Steroids (7/8), RTX (5/8), bortezomib (4/8), IVIg (4/8), PE (2/8), MMF (4/8), No treatment (1/8), vincristine (1/8), cyclophosphamide (1/8)	One patient failed 8 modes of therapy and died of persistent refractory pancytopenia and multiorgan failure 144 days post-UCBT	Multivariable analysis identified ALC as the most significant predictor of IMC in this population (adjusted odds ratio, 2.186; 95% confidence interval, 1.047–4.559; P = .037) IMC is a result of failed suppression of recipient immunity and part of a spectrum including graft rejection
Szanto et al. [15]	380 pediatric patients receiving HCT	AIHA, ITP, neutropenia alone or in combination; 30/380 patients (incidence of IMCs after HCT of 7.8%)	21/30 non-malignant diseases	Steroids (all patients), MMF (15/30) sirolimus (12/30) RTX (19/30)	4/30 responded to first-line therapy; overall all patients responded to additional therapies; OS 83%	In multivariate Cox regression analysis, aGVHD grades II to IV (hazard ratio [HR], 2.45; 95% confidence interval [CI], 1.18 to 5.09; *P* = .0167), chemo-naivety before HCT (HR, 2.36; 95% CI, 1.00 to 5.57; *P* = .0499), and serotherapy (HR, 8.00; 95% CI, 1.05 to 61.04; *P* = .045) were independent predictors for AIC development
Galvin et al. [25]	297 pediatric patients receiving HCT for NMD	AIHA, ITP, neutropenia alone or in combination; 50/297 patients developed IMC (cumulative incidence of 18.4%)	50/50 Non-malignant diseases: 26/50 Inherited metabolic disorder; 6/50 Hemoglobinopathy; 8/50 SAA; 6/50 Primary immune deficiency; 4/50 Epidermolysis bullosa	90% of cases required at least supportive care, those with intermediate or prolonged IMC, all received corticosteroid therapy, with a response rate of 34%. The remainder required additional treatment (IVIGs, RTX, bortezomib, PE)	In the 50 patients with IMC, 6 died; 4 were attributable to IMC (8%). There was no difference in overall survival between those with IMC and without	Fine-Gray competing risk multivariate regression analysis identified a combined risk factor of younger age (< 3 years) and inherited metabolic disorder, as well as hemoglobinopathy (at any age) associated with 1-year incidence of IMC (*P* < .01)

*AD* autoimmune diseases, *AHIA* autoimmune hemolytic anemia, *BDS* Blackfan-Diamond syndrome, *FA* Fanconi anemia, *CA* congenital anemia, *SCID* severe combined immunodeficiency disease, *SCAEBV* severe chronic active EBV infection syndrome, *WAS* Wiskott-Aldrich syndrome; *MP* methyl prednisone, *dex* pulse dexamethasone, *HLH* hemophagocytic lymphohistiocytosis, *IVIg* intravenous immunoglobulin, *ITP* immune thrombocytopenia, *Langh His* Langerhans cell histiocytosis, *MPS1*, mucopolysaccharidosis type 1, *CSA* ciclosporin, *RTX* rituximab, *PE* plasmapheresis, *MMF* mycophenolate, *EPO* erythropoietin, *SAA* severe aplastic anemia

1. O’Brien TA, Eastlund T, Peters C, Neglia JP, Defer T, Ramsay NKC, et al. Autoimmune haemolytic anaemia complicating haematopoietic cell transplantation in paediatric patients: high incidence and significant mortality in unrelated donor transplants for non-malignant diseases. Br J Haematol. 2004;

2. Page KM, Mendizabal AM, Prasad VK, Martin PL, Parikh S, Wood S, et al. Posttransplant autoimmune hemolytic anemia and other autoimmune cytopenias are increased in very young infants undergoing unrelated donor umbilical cord blood transplantation. Biol Blood Marrow Transplant [Internet]. 2008 Oct [cited 2022 Jan 9];14(10):1108–17. Available from: https://pubmed.ncbi.nlm.nih.gov/18804040/

3. Daikeler T, Labopin M, Ruggeri A, Crotta A, Abinun M, Hussein AA, et al. New autoimmune diseases after cord blood transplantation: a retrospective study of EUROCORD and the autoimmune disease working party of the European group for blood and marrow transplantation. Blood. 2013;

4. Faraci M, Zecca M, Pillon M, Rovelli A, Menconi MC, Ripaldi M, et al. Autoimmune hematological diseases after allogeneic hematopoietic stem cell transplantation in children: an Italian multicenter experience. Biol Blood Marrow Transplant. 2014;

5. Ahmed I, Teruya J, Murray-Krezan C, Krance R. The incidence of autoimmune hemolytic anemia in pediatric hematopoietic stem cell recipients post-first and post-second hematopoietic stem cell transplant. Pediatr Transplant [Internet]. 2015 Jun 1 [cited 2022 Jan 9];19(4):391–8. Available from: https://pubmed.ncbi.nlm.nih.gov/25809012/

6. Kruizinga MD, van Tol MJD, Bekker V, Netelenbos T, Smiers FJ, Bresters D, et al. Risk factors, treatment, and immune dysregulation in autoimmune cytopenia after allogeneic hematopoietic stem cell transplantation in pediatric patients. Biol Blood Marrow Transplant [Internet]. 2018 Apr 1 [cited 2022 Jan 8];24(4):772–8. Available from: https://pubmed.ncbi.nlm.nih.gov/29277513/

7. Deambrosis D, Lum SH, Hum RM, Poulton K, Ogden W, Jones S, et al. Immune cytopenia post-cord transplant in Hurler syndrome is a forme fruste of graft rejection. Blood Adv [Internet]. 2019 Feb 26 [cited 2022 Jan 8];3(4):570–4. Available from: https://pubmed.ncbi.nlm.nih.gov/30787020/

8. Szanto CL, Langenhorst J, de Koning C, Nierkens S, Bierings M, Huitema ADR, et al. Predictors for autoimmune cytopenias after allogeneic hematopoietic cell transplantation in children. Biol Blood Marrow Transplant [Internet]. 2020 Jan 1 [cited 2022 Jan 9];26(1):114–22. Available from: https://pubmed.ncbi.nlm.nih.gov/31344451/

9. Galvin RT, Cao Q, Miller WP, Knight-Perry J, Smith AR, Ebens CL. Characterizing immune-mediated cytopenias after allogeneic hematopoietic cell transplantation for pediatric nonmalignant disorders. Transplant Cell Ther. 2021 Apr 1;27(4):316.e1-316.e8

## Expert opinion

Notwithstanding the difficulties in drawing conclusions from the available data, the authors suggest that some unique patient subgroups (e.g., those expected as refractory to standard therapies) could benefit from a prompt second- or even third-line treatment based on the newest drugs, even if their potential effectiveness deserves to be further assessed, so far. Indeed, patients presenting mixed chimerism should receive DLI or a second HSCT, either if affected by hemolytic anemia or immune thrombocytopenia. For young patients with hemolytic anemia after HCT and concomitant GvHD, a good choice could be to administer drugs that could indirectly help manage the GvHD itself: ibrutinib or bortezomib could be the best option, from this standpoint. Ultimately, for patients with immune thrombocytopenia and GvHD, low-dose IL2 or fostamatinib could represent a valid therapeutic approach. A careful risk/benefit balance should always guide the clinician’s decision.

## Conclusion

IMCs arising in the post-HSCT setting represent a rare but potentially life-threatening complication. It is widely reported that younger age at transplantation carries a higher risk of IMCs post-HSCT, especially considering young patients transplanted for non-malignant disorders. Moreover, as compared to adults, children present unique risk factors which could be interpreted as the result of the complex relationship between the immaturity of their infantile immune system and all the perturbing agents and factors which characterize the post-HSCT period. Corticosteroids, intravenous immunoglobulin, and rituximab represent the undiscussed first-line therapeutic approach, whereas future efforts should be directed to establish the best option for refractory patients, for whom a standard and validated approach is not currently available. Notwithstanding this uncertain scenario, the last decade has witnessed an improvement in the management of these challenging immune diseases, but further studies are warranted to offer a better outcome for these fragile patients.

## Abbreviations

aGvHD: Acute graft-versus-host disease
BM: Bone marrow
BTK: Bruton’s tyrosine kinase
cGvHD: Chronic graft-versus-host disease
CLL: Chronic lymphocytic leukemia
CR: Complete response
DAT: Direct antiglobulin test
FcRn: Neonatal crystallizable fragment receptor
G-CSF: Granulocyte colony-stimulating factor
GvHD: Graft-versus-host disease
HSCT: Hematopoietic stem cell transplantation
IMCs: Immune-mediated cytopenias
ORR: Overall response rates
PBSC: Peripheral blood stem cells
Th2: T helper cell type 2
UCB: Unrelated cord blood
